# (6*S*,7*S*,8*R*,8a*S*)-6-Ethyl­perhydro­indolizine-7,8-diol

**DOI:** 10.1107/S1600536810021240

**Published:** 2010-06-16

**Authors:** Ľubomír Švorc, Viktor Vrábel, Jozefína Žúžiová, Štefan Marchalín, Jozef Kožíšek

**Affiliations:** a Institute of Analytical Chemistry, Faculty of Chemical and Food Technology, Slovak Technical University, Radlinského 9, SK-812 37 Bratislava, Slovak Republic 81237; b Institute of Organic Chemistry, Catalysis and Petrochemistry, Faculty of Chemical and Food Technology, Slovak Technical University, Radlinského 9, SK-812 37 Bratislava, Slovak Republic 81237; c Institute of Physical Chemistry and Chemical Physics, Faculty of Chemical and Food Technology, Slovak Technical University, Radlinského 9, Bratislava, Slovak Republic 81237

## Abstract

In the title compound, C_10_H_19_NO_2_, the piperidine and pyrrolidine rings of the perhydro­indolizine ring system adopt chair and envelope conformations, respectively. In the crystal structure, inter­molecular O—H⋯N and O—H⋯O hydrogen bonds link the mol­ecules into a chain running along the *a* axis.

## Related literature

For indolizine derivatives, see: Bermudez *et al.* (1990 ▶); Bonneau *et al.* (2003 ▶); Chai *et al.* (2003 ▶); Delattre *et al.* (2005 ▶); Gundersen *et al.* (2007 ▶); Liu *et al.* (2007 ▶); Teklu *et al.* (2005 ▶); Weide *et al.* (2006 ▶). For ring conformations, see: Cremer & Pople (1975 ▶); Nardelli (1983 ▶). For the synthesis, see: Šafař *et al.* (2010 ▶). 
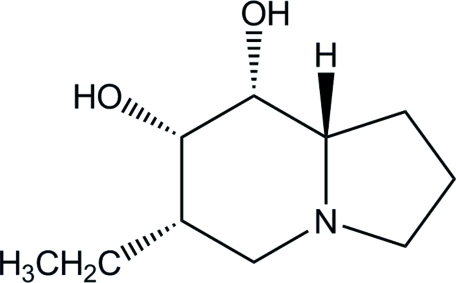


## Experimental

### 

#### Crystal data

C_10_H_19_NO_2_
*M* *_r_* = 185.26
Orthorhombic, 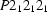

*a* = 7.20849 (17) Å
*b* = 8.83039 (19) Å
*c* = 15.6656 (4) Å
*V* = 997.18 (4) Å^3^
*Z* = 4
Mo *K*α radiation
μ = 0.09 mm^−1^
*T* = 298 K
0.51 × 0.29 × 0.09 mm

#### Data collection

Oxford Diffraction Gemini R CCD diffractometer
Absorption correction: analytical (Clark & Reid, 1995 ▶) *T*_min_ = 0.950, *T*_max_ = 0.992
26407 measured reflections
1554 independent reflections
1371 reflections with *I* > 2σ(*I*)
*R*_int_ = 0.023

#### Refinement

*R*[*F* ^2^ > 2σ(*F* ^2^)] = 0.035
*wR*(*F* ^2^) = 0.099
*S* = 1.07
1554 reflections
124 parameters
H atoms treated by a mixture of independent and constrained refinement
Δρ_max_ = 0.17 e Å^−3^
Δρ_min_ = −0.18 e Å^−3^

### 

Data collection: *CrysAlis CCD* (Oxford Diffraction, 2006 ▶); cell refinement: *CrysAlis RED* (Oxford Diffraction, 2006 ▶); data reduction: *CrysAlis RED*; program(s) used to solve structure: *SHELXS97* (Sheldrick, 2008 ▶); program(s) used to refine structure: *SHELXL97* (Sheldrick, 2008 ▶); molecular graphics: *DIAMOND* (Brandenburg, 2001 ▶); software used to prepare material for publication: *enCIFer* (Allen *et al.*, 2004 ▶) and *PLATON* (Spek, 2009 ▶).

## Supplementary Material

Crystal structure: contains datablocks I, global. DOI: 10.1107/S1600536810021240/is2552sup1.cif

Structure factors: contains datablocks I. DOI: 10.1107/S1600536810021240/is2552Isup2.hkl

Additional supplementary materials:  crystallographic information; 3D view; checkCIF report

## Figures and Tables

**Table 1:** Hydrogen-bond geometry (Å, °)

*D*—H⋯*A*	*D*—H	H⋯*A*	*D*⋯*A*	*D*—H⋯*A*
O1—H1*A*⋯N1^i^	0.79 (2)	2.104 (19)	2.8619 (16)	160.2 (18)
O12—H12*A*⋯O1^i^	0.82 (2)	2.05 (2)	2.8591 (15)	169 (2)

Symmetry code: (i) 
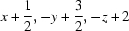
.

**Fig. 1.**
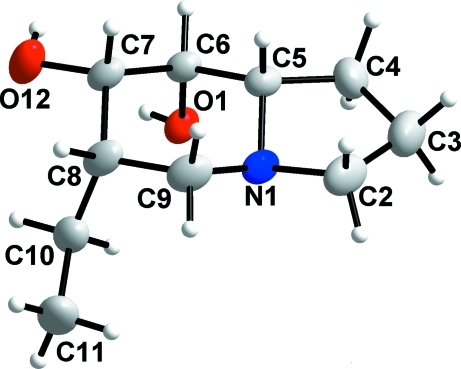
Molecular structure of (I) with the atomic numbering scheme. Displacement ellipsoids are drawn at the 50% probability level.

**Fig. 2.**
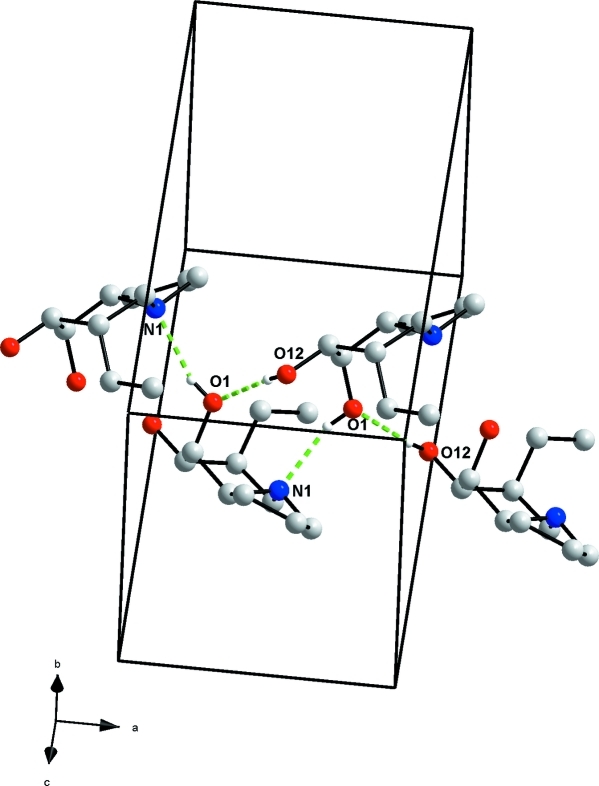
A partial packing diagram of the molecule of (I), showing a molecular chain along the a axis. Hydrogen bonds are indicated by dashed lines. H atoms not involved in the hydrogen bonds have been omitted.

## supplementary crystallographic information

### Comment

Bridgehead nitrogen heterocycles are important natural products. Among them, indolizines have received much attention in recent years due to their intriguing molecular structures featured with a 10 *p*-delocalized electrons. They have been extensively examined because of its wide range of potent applications such as biological activities (Bonneau *et al.*, 2003) and a fluorescent probe (Delattre *et al.*, 2005). These molecules have found various pharmaceutical applications as anti-tuberculosis agents (Gundersen *et al.*, 2007), histamine H3 receptor antagonists (Chai *et al.*, 2003), 5-HT3 receptor antagonists (Bermudez *et al.*, 1990), associated with many infectious diseases (Weide *et al.*, 2006) and as 15-lipoxygenase inhibitors (Teklu *et al.*, 2005). Indolizines demonstrate also antifungal, antimycobacterial, antiherpes and antineociceptive properties (Liu *et al.*, 2007). Thus, there is a growing interest in the synthesis and study of crystal and molecular structures of indolizine derivatives.

Based on these facts and in continuation of our interest in developing simple and efficient route for the synthesis of novel monohydroxylated indolizine derivatives, we report here the synthesis, molecular and crystal structure of the title compound, (I). The absolute configuration was established by synthesis and is depicted in the scheme and figure. The expected stereochemistry of atoms C5, C6, C7 and C8 was confirmed as S, R, S and S, respectively (Fig. 1). The central six-membered ring is not planar and adopts a chair conformation (Cremer & Pople, 1975). A calculation of least-squares planes shows that this ring is puckered in such a manner that the four atoms C6, C7, C9 and N1 are coplanar to within 0.019 (2) Å, while atoms C5 and C8 are displaced from this plane on opposite sides, with out-of-plane displacements of -0.720 (2) and 0.636 (1) Å, respectively. In the molecule, the pyrrolidine ring N1/C2–C5 exhibits an envelope conformation with envelope on atom N1 (Nardelli, 1983). The displacement of atom N1 from the mean plane of the remaining four atoms is 0.625 (2) Å. The N1—C2, N1—C5 and N1—C9 bonds are approximately equivalent. Atom N1 is sp^3^-hybridized, as evidenced by the sum of the valence angles around it [327.05 (2)°]. Intermolecular O—H···N and O—H···O hydrogen bonds link the neighbouring molecules of (I) into extended chains, which run parallel to the a axis (Fig. 2) and help to stabilize the crystal structure of the compound. Atom N1 (O1) participates as acceptor and atom O1 (O12) as donator in these intermolecular hydrogen bonds.

### Experimental

The title compound (6*S*,7*S*,8*R*,8aS)-6-ethylperhydroindolizine-7,8-diol was prepared according literature procedures of Šafař *et al.* (2010).

### Refinement

Hydroxyl H atoms were located in a difference Fourier map and their positions were refined freely, with *U*_iso_(H) = 1.2*U*_eq_(O). Other H atoms were placed in geometrically idealized positions and constrained to ride on their parent atoms, with C—H distances in the range 0.93–0.98 Å, and with *U*_iso_(H) = 1.2*U*_eq_(C). The absolute configuration could not be reliably determined for this compound using Mo radiation, and has been assigned according to the synthesis. 1061 total Friedel pairs have been merged.

### Crystal data

**Table:**

C_10_H_19_NO_2_	*F*(000) = 408
*M**_r_* = 185.26	*D*_x_ = 1.234 Mg m^−^^3^
Orthorhombic, *P*2_1_2_1_2_1_	Mo *K*α radiation, λ = 0.71073 Å
Hall symbol: P 2ac 2ab	Cell parameters from 17389 reflections
*a* = 7.20849 (17) Å	θ = 3.5–29.5°
*b* = 8.83039 (19) Å	µ = 0.09 mm^−^^1^
*c* = 15.6656 (4) Å	*T* = 298 K
*V* = 997.18 (4) Å^3^	Prism, white
*Z* = 4	0.51 × 0.29 × 0.09 mm

### Data collection

**Table:**

Oxford Diffraction Gemini R CCD diffractometer	1554 independent reflections
Radiation source: fine-focus sealed tube	1371 reflections with *I* > 2σ(*I*)
graphite	*R*_int_ = 0.023
Detector resolution: 10.4340 pixels mm^-1^	θ_max_ = 29.5°, θ_min_ = 3.5°
Rotation method data acquisition using ω and φ scans	*h* = −9→9
Absorption correction: analytical (Clark & Reid, 1995)	*k* = −11→12
*T*_min_ = 0.950, *T*_max_ = 0.992	*l* = −21→20
26407 measured reflections	

### Refinement

**Table:**

Refinement on *F*^2^	Primary atom site location: structure-invariant direct methods
Least-squares matrix: full	Secondary atom site location: difference Fourier map
*R*[*F*^2^ > 2σ(*F*^2^)] = 0.035	Hydrogen site location: inferred from neighbouring sites
*wR*(*F*^2^) = 0.099	H atoms treated by a mixture of independent and constrained refinement
*S* = 1.07	*w* = 1/[σ^2^(*F*_o_^2^) + (0.0669*P*)^2^ + 0.0442*P*] where *P* = (*F*_o_^2^ + 2*F*_c_^2^)/3
1554 reflections	(Δ/σ)_max_ < 0.001
124 parameters	Δρ_max_ = 0.17 e Å^−^^3^
0 restraints	Δρ_min_ = −0.18 e Å^−^^3^

### Special details

**Table:**

Experimental. (face-indexed; Oxford Diffraction, 2006)
Geometry. All esds (except the esd in the dihedral angle between two l.s. planes) are estimated using the full covariance matrix. The cell esds are taken into account individually in the estimation of esds in distances, angles and torsion angles; correlations between esds in cell parameters are only used when they are defined by crystal symmetry. An approximate (isotropic) treatment of cell esds is used for estimating esds involving l.s. planes.
Refinement. Refinement of *F*^2^ against ALL reflections. The weighted *R*-factor wR and goodness of fit *S* are based on *F*^2^, conventional *R*-factors *R* are based on *F*, with *F* set to zero for negative *F*^2^. The threshold expression of *F*^2^ > σ(*F*^2^) is used only for calculating *R*-factors(gt) etc. and is not relevant to the choice of reflections for refinement. *R*-factors based on *F*^2^ are statistically about twice as large as those based on *F*, and *R*- factors based on ALL data will be even larger.

### Fractional atomic coordinates and isotropic or equivalent isotropic displacement parameters (Å^2^)

**Table:**

	*x*	*y*	*z*	*U*_iso_*/*U*_eq_	
C2	0.3886 (2)	0.5154 (2)	0.85244 (11)	0.0478 (4)	
H2B	0.2624	0.5248	0.8732	0.057*	
H2A	0.4043	0.4161	0.8270	0.057*	
C3	0.4326 (2)	0.6396 (3)	0.78828 (12)	0.0544 (5)	
H3B	0.3442	0.7220	0.7931	0.065*	
H3A	0.4284	0.6003	0.7305	0.065*	
C4	0.6291 (2)	0.6945 (2)	0.81031 (10)	0.0424 (4)	
H4B	0.7087	0.6928	0.7604	0.051*	
H4A	0.6266	0.7964	0.8334	0.051*	
C5	0.69456 (19)	0.58078 (16)	0.87717 (9)	0.0311 (3)	
H5A	0.7393	0.4907	0.8470	0.037*	
C6	0.84249 (18)	0.62711 (14)	0.94069 (9)	0.0277 (3)	
H6A	0.9566	0.6503	0.9093	0.033*	
C7	0.8790 (2)	0.49092 (15)	0.99892 (9)	0.0314 (3)	
H7A	0.9272	0.4098	0.9624	0.038*	
C8	0.7028 (2)	0.42864 (16)	1.04186 (10)	0.0346 (3)	
H8A	0.7353	0.3283	1.0640	0.042*	
C9	0.5529 (2)	0.40321 (17)	0.97423 (11)	0.0393 (4)	
H9B	0.5893	0.3199	0.9375	0.047*	
H9A	0.4376	0.3756	1.0021	0.047*	
C10	0.6369 (2)	0.52143 (19)	1.11850 (10)	0.0401 (4)	
H10B	0.7403	0.5354	1.1573	0.048*	
H10A	0.5987	0.6207	1.0987	0.048*	
C11	0.4772 (3)	0.4499 (3)	1.16706 (12)	0.0624 (6)	
H11C	0.4429	0.5140	1.2140	0.075*	
H11B	0.5147	0.3527	1.1884	0.075*	
H11A	0.3730	0.4377	1.1295	0.075*	
N1	0.52295 (16)	0.53891 (14)	0.92216 (8)	0.0319 (3)	
O1	0.78542 (14)	0.75959 (11)	0.98549 (6)	0.0301 (2)	
H1A	0.869 (3)	0.7981 (19)	1.0102 (12)	0.036*	
O12	1.01533 (16)	0.51868 (14)	1.06187 (8)	0.0450 (3)	
H12A	1.085 (3)	0.582 (2)	1.0417 (14)	0.054*	

### Atomic displacement parameters (Å^2^)

**Table:**

	*U*^11^	*U*^22^	*U*^33^	*U*^12^	*U*^13^	*U*^23^
C2	0.0338 (7)	0.0584 (10)	0.0512 (9)	−0.0026 (8)	−0.0100 (7)	−0.0214 (8)
C3	0.0466 (9)	0.0696 (12)	0.0471 (9)	0.0076 (9)	−0.0156 (8)	−0.0088 (9)
C4	0.0475 (9)	0.0487 (8)	0.0310 (7)	0.0031 (8)	−0.0053 (7)	−0.0016 (6)
C5	0.0280 (6)	0.0318 (6)	0.0334 (6)	0.0016 (6)	0.0015 (5)	−0.0074 (5)
C6	0.0237 (6)	0.0254 (6)	0.0341 (6)	−0.0005 (5)	0.0016 (5)	−0.0009 (5)
C7	0.0242 (6)	0.0264 (6)	0.0437 (8)	0.0013 (5)	0.0007 (5)	0.0006 (5)
C8	0.0282 (7)	0.0241 (6)	0.0515 (8)	0.0003 (6)	0.0022 (6)	0.0072 (6)
C9	0.0323 (7)	0.0281 (7)	0.0575 (9)	−0.0072 (6)	0.0030 (7)	−0.0038 (6)
C10	0.0360 (7)	0.0458 (8)	0.0385 (7)	0.0043 (7)	0.0019 (6)	0.0102 (6)
C11	0.0378 (8)	0.0995 (16)	0.0500 (9)	0.0001 (10)	0.0066 (8)	0.0219 (11)
N1	0.0242 (5)	0.0326 (6)	0.0387 (6)	−0.0031 (5)	−0.0028 (5)	−0.0084 (5)
O1	0.0274 (5)	0.0244 (4)	0.0385 (5)	−0.0001 (4)	−0.0056 (4)	−0.0038 (4)
O12	0.0305 (6)	0.0498 (7)	0.0548 (7)	−0.0060 (5)	−0.0097 (5)	0.0156 (6)

### Geometric parameters (Å, °)

**Table:**

C2—N1	1.4742 (18)	C7—C8	1.5386 (19)
C2—C3	1.521 (3)	C7—H7A	0.9800
C2—H2B	0.9700	C8—C10	1.529 (2)
C2—H2A	0.9700	C8—C9	1.530 (2)
C3—C4	1.536 (2)	C8—H8A	0.9800
C3—H3B	0.9700	C9—N1	1.465 (2)
C3—H3A	0.9700	C9—H9B	0.9700
C4—C5	1.526 (2)	C9—H9A	0.9700
C4—H4B	0.9700	C10—C11	1.518 (2)
C4—H4A	0.9700	C10—H10B	0.9700
C5—N1	1.4710 (17)	C10—H10A	0.9700
C5—C6	1.5148 (18)	C11—H11C	0.9600
C5—H5A	0.9800	C11—H11B	0.9600
C6—O1	1.4250 (16)	C11—H11A	0.9600
C6—C7	1.5322 (18)	O1—H1A	0.79 (2)
C6—H6A	0.9800	O12—H12A	0.82 (2)
C7—O12	1.4137 (18)		
			
N1—C2—C3	104.54 (13)	O12—C7—H7A	106.7
N1—C2—H2B	110.8	C6—C7—H7A	106.7
C3—C2—H2B	110.8	C8—C7—H7A	106.7
N1—C2—H2A	110.8	C10—C8—C9	113.74 (12)
C3—C2—H2A	110.8	C10—C8—C7	114.10 (12)
H2B—C2—H2A	108.9	C9—C8—C7	109.43 (12)
C2—C3—C4	105.74 (14)	C10—C8—H8A	106.3
C2—C3—H3B	110.6	C9—C8—H8A	106.3
C4—C3—H3B	110.6	C7—C8—H8A	106.3
C2—C3—H3A	110.6	N1—C9—C8	111.68 (11)
C4—C3—H3A	110.6	N1—C9—H9B	109.3
H3B—C3—H3A	108.7	C8—C9—H9B	109.3
C5—C4—C3	103.41 (15)	N1—C9—H9A	109.3
C5—C4—H4B	111.1	C8—C9—H9A	109.3
C3—C4—H4B	111.1	H9B—C9—H9A	107.9
C5—C4—H4A	111.1	C11—C10—C8	113.97 (15)
C3—C4—H4A	111.1	C11—C10—H10B	108.8
H4B—C4—H4A	109.0	C8—C10—H10B	108.8
N1—C5—C6	110.19 (11)	C11—C10—H10A	108.8
N1—C5—C4	103.54 (12)	C8—C10—H10A	108.8
C6—C5—C4	119.40 (13)	H10B—C10—H10A	107.7
N1—C5—H5A	107.7	C10—C11—H11C	109.5
C6—C5—H5A	107.7	C10—C11—H11B	109.5
C4—C5—H5A	107.7	H11C—C11—H11B	109.5
O1—C6—C5	110.00 (11)	C10—C11—H11A	109.5
O1—C6—C7	113.62 (11)	H11C—C11—H11A	109.5
C5—C6—C7	107.46 (11)	H11B—C11—H11A	109.5
O1—C6—H6A	108.5	C9—N1—C5	110.39 (11)
C5—C6—H6A	108.5	C9—N1—C2	113.21 (12)
C7—C6—H6A	108.5	C5—N1—C2	103.45 (11)
O12—C7—C6	113.49 (11)	C6—O1—H1A	112.0 (13)
O12—C7—C8	109.31 (11)	C7—O12—H12A	106.1 (15)
C6—C7—C8	113.52 (12)		
			
N1—C2—C3—C4	18.32 (17)	O12—C7—C8—C9	178.22 (11)
C2—C3—C4—C5	8.30 (17)	C6—C7—C8—C9	50.40 (15)
C3—C4—C5—N1	−32.09 (15)	C10—C8—C9—N1	76.70 (16)
C3—C4—C5—C6	−154.99 (13)	C7—C8—C9—N1	−52.21 (16)
N1—C5—C6—O1	−63.72 (14)	C9—C8—C10—C11	60.38 (17)
C4—C5—C6—O1	55.85 (16)	C7—C8—C10—C11	−173.12 (12)
N1—C5—C6—C7	60.44 (14)	C8—C9—N1—C5	60.58 (15)
C4—C5—C6—C7	−179.99 (12)	C8—C9—N1—C2	176.00 (12)
O1—C6—C7—O12	−58.02 (16)	C6—C5—N1—C9	−65.22 (14)
C5—C6—C7—O12	−179.95 (11)	C4—C5—N1—C9	165.98 (11)
O1—C6—C7—C8	67.60 (15)	C6—C5—N1—C2	173.37 (12)
C5—C6—C7—C8	−54.33 (14)	C4—C5—N1—C2	44.57 (14)
O12—C7—C8—C10	49.50 (16)	C3—C2—N1—C9	−158.49 (13)
C6—C7—C8—C10	−78.32 (15)	C3—C2—N1—C5	−39.00 (16)

### Hydrogen-bond geometry (Å, °)

**Table:**

*D*—H···*A*	*D*—H	H···*A*	*D*···*A*	*D*—H···*A*
O1—H1A···N1^i^	0.79 (2)	2.104 (19)	2.8619 (16)	160.2 (18)
O12—H12A···O1^i^	0.82 (2)	2.05 (2)	2.8591 (15)	169 (2)

Symmetry codes: (i) *x*+1/2, −*y*+3/2, −*z*+2.
